# Inferring ancestral range reconstruction based on trilobite records: a study-case on *Metacryphaeus* (Phacopida, Calmoniidae)

**DOI:** 10.1038/s41598-018-33517-5

**Published:** 2018-10-12

**Authors:** Fábio Augusto Carbonaro, Max Cardoso Langer, Silvio Shigueo Nihei, Gabriel de Souza Ferreira, Renato Pirani Ghilardi

**Affiliations:** 1 Departamento de Ciências Biológicas, Faculdade de Ciências de Bauru, Universidade Estadual Paulista Júlio de Mesquita Filho – UNESP, Av. Eng. Luiz Edmundo Carrijo Coube, 14-01, CEP 17033-360 Bauru/SP, Brazil; 2 Laboratório de Paleontologia, Faculdade de Filosofia, Ciências e Letras de Ribeirão Preto, Universidade de São Paulo – USP, Av. Bandeirantes, 3900, CEP 14040-901 Ribeirão Preto/SP, Brazil; 3 Departamento de Zoologia, Instituto de Biociências, Universidade de São Paulo – USP, Rua do Matão, Travessa 14, 101, Butantã, CEP 05508-090 São Paulo/SP, Brazil; 4 Fachbereich Geowissenschaften der Eberhard Karls Universität Tübingen, Hölderlinstraße 12, 72074 Tübingen, Germany

## Abstract

*Metacryphaeus* is a calmoniid trilobite genus from the Devonian Malvinokaffric Realm, exclusive to the Gondwanan regions. It includes eleven species, which are for the first time included here in a single phylogenetic analysis. The resulting hypotheses establish relations among the *Metacryphaeus* species with few ambiguities, also suggesting the inclusion of both *Plesiomalvinella pujravii* and *P. boulei* within the genus, as originally considered. The results of palaeobiogeographic analyses employing the Dispersal-Extinction-Cladogenesis (DEC) model reinforce the hypothesis that Bolivia and Peru form the ancestral home of *Metacryphaeus*. The radiation of the genus to other Gondwanan areas took place during transgressive eustatic episodes during the Lochkovian–Pragian. The Lochkovian dispersal occurred from Bolivia and Peru to Brazil (Paraná and Parnaíba basins) and the Falklands, and Pragian dispersal occurred towards South Africa. Dispersal events from Bolivia and Peru to the Parnaíba Basin (Brazil) were identified during the Lochkovian–Pragian, suggesting the presence of marine connections between those areas earlier than previously thought.

## Introduction

The Malvinokaffric Realm includes a plethora of trilobites, including the Calmoniidae, which is composed of several genera (*e.g*., *Calmonia* Clarke, 1913, *Typhloniscus* Salter, 1856, *Plesioconvexa* Lieberman, 1993, *Punillaspis* Baldis & Longobucco, 1977, *Eldredgeia* Lieberman, 1993, *Clarkeaspis* Lieberman, 1993, *Malvinocooperella* Lieberman, 1993, *Wolfartaspis* Cooper, 1982, *Metacryphaeus* Reed, 1907) reported from the Devonian rocks of Brazil, Argentina, Bolivia, Peru, Falkland Islands, and South Africa^1–10^. The present work focuses on the genus *Metacryphaeus*, which only includes Gondwanan species, namely: *M. tuberculatus* (Kozłowski, 1923), *M. kegeli* Carvalho *et al*., 1997, *M. meloi* Carvalho *et al*., 1997, *M. parana* (Kozłowski, 1923), *M. giganteus* (Ulrich, 1892), *M. convexus* (Ulrich, 1892), *M. curvigena* Lieberman, 1993, *M. branisai* Lieberman, 1993, *M. caffer* (Salter, 1856), *M. australis* (Clarke, 1913), and *M. allardyceae* (Clarke, 1913).

**Figure 1:**
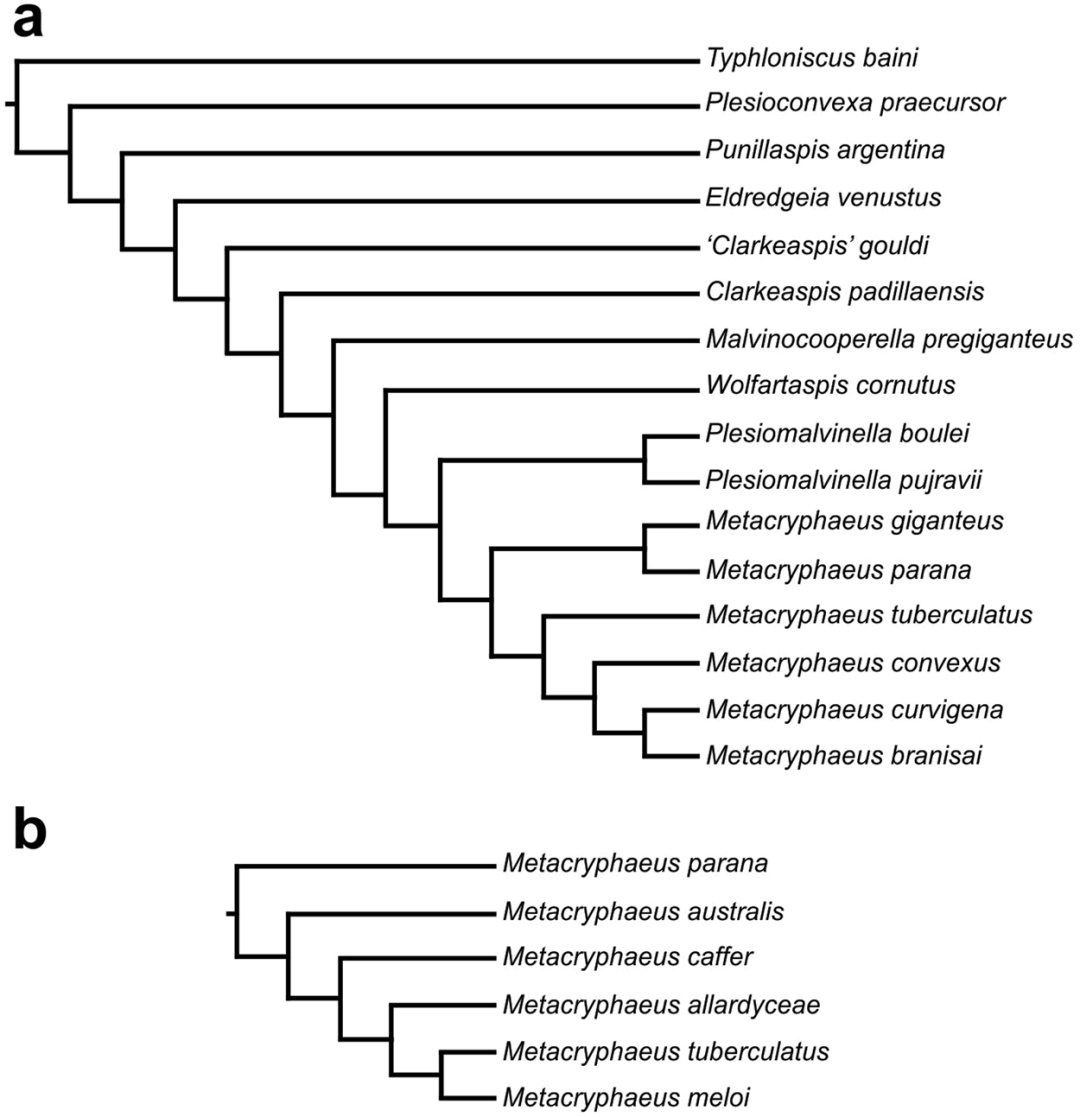
Previous phylogenetic models including *Metacryphaeus*: (**a**) Lieberman^5^; and (**b**) Carvalho *et al*.^6^.

During the 1990s, Lieberman^5^ presented the first phylogenetic analysis of the group including *Metacryphaeus*, represented by *M. parana*, *M. convexus*, *M. curvigena*, *M. branisai*, *M. giganteus*, and *M. tuberculatus*, among other calmoniids (Fig. 1a). Later, Carvalho *et al*.^6^ (Fig. 1b) conducted a phylogenetic study of that genus, represented by *M. parana*, *M. australis*, *M. caffer*, *M. allardyceae*, *M. tuberculatus*, and *M. meloi*. More recently, Abe & Lieberman^9^ presented a palaeobiogeographical area-taxon cladogram including all *Metacryphaeus* species, based on the tree provided by Lieberman^5^, with the manual insertion of additional species (i.e., without carrying out a new phylogenetic analysis). Those phylogenetic studies did not include all the species of *Metacryphaeus*. Accordingly, this study provides a new phylogenetic analysis including all species, in order to perform a new palaeobiogeographic analysis for the distribution of the genus.

**Figure 2:**
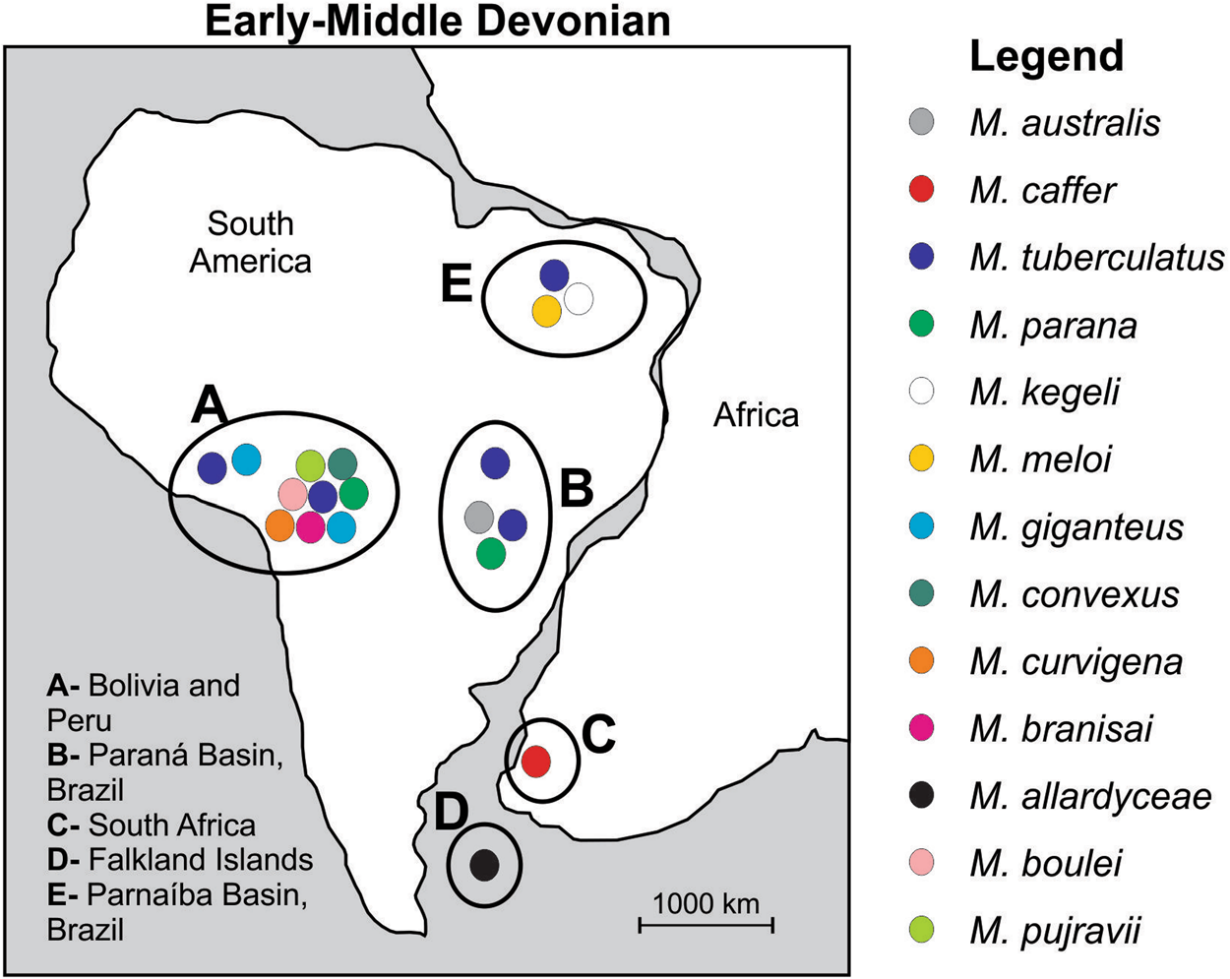
Palaeobiogeographic distribution of the genus *Metacryphaeus* during the Early and Middle Devonian. Areas were divided into (A–E) for the palaeobiogeographic analysis. Note: *Plesiomalvinella boulei* and *P. pujravii* have been reassigned here to *Metacryphaeus*.

**Figure 3:**
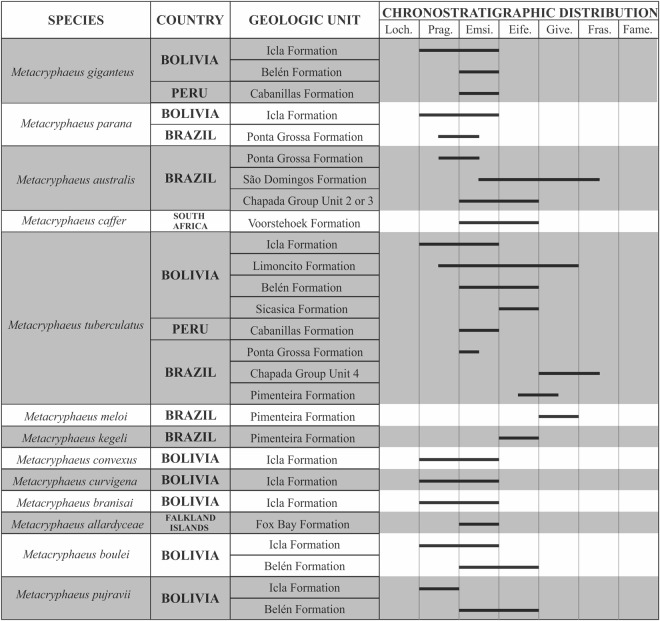
Chronostratigraphic distribution of *Metacryphaeus*. Note: *Plesiomalvinella boulei* and *P. pujravii* have been reassigned here to *Metacryphaeus*. Abbreviations: Loch., Lochkovian; Prag., Pragian; Emsi., Emsian; Eife., Efelian; Give., Givetian; Fras., Frasnian; Fame., Famennian.

The genus *Metacryphaeus* occurs in many Gondwanan geological units of Devonian age, including those in Brazil, Bolivia, Falkland Islands, Peru, and South Africa, spanning the Pragian to the Givetian–Frasnian^3–6,11,12^ (Figs 2 and 3). It has been suggested that the genus originated and diversified in small basins of the Malvinokaffric Realm in Bolivia and Peru^9^. The records in this area are from the Pragian to the Givetian, including *M. giganteus*, *M. tuberculatus*, *M. parana*, *M. convexus*, *M. curvigena*, and *M. branisai*.

## Results and Discussion

### Phylogeny

**Figure 4:**
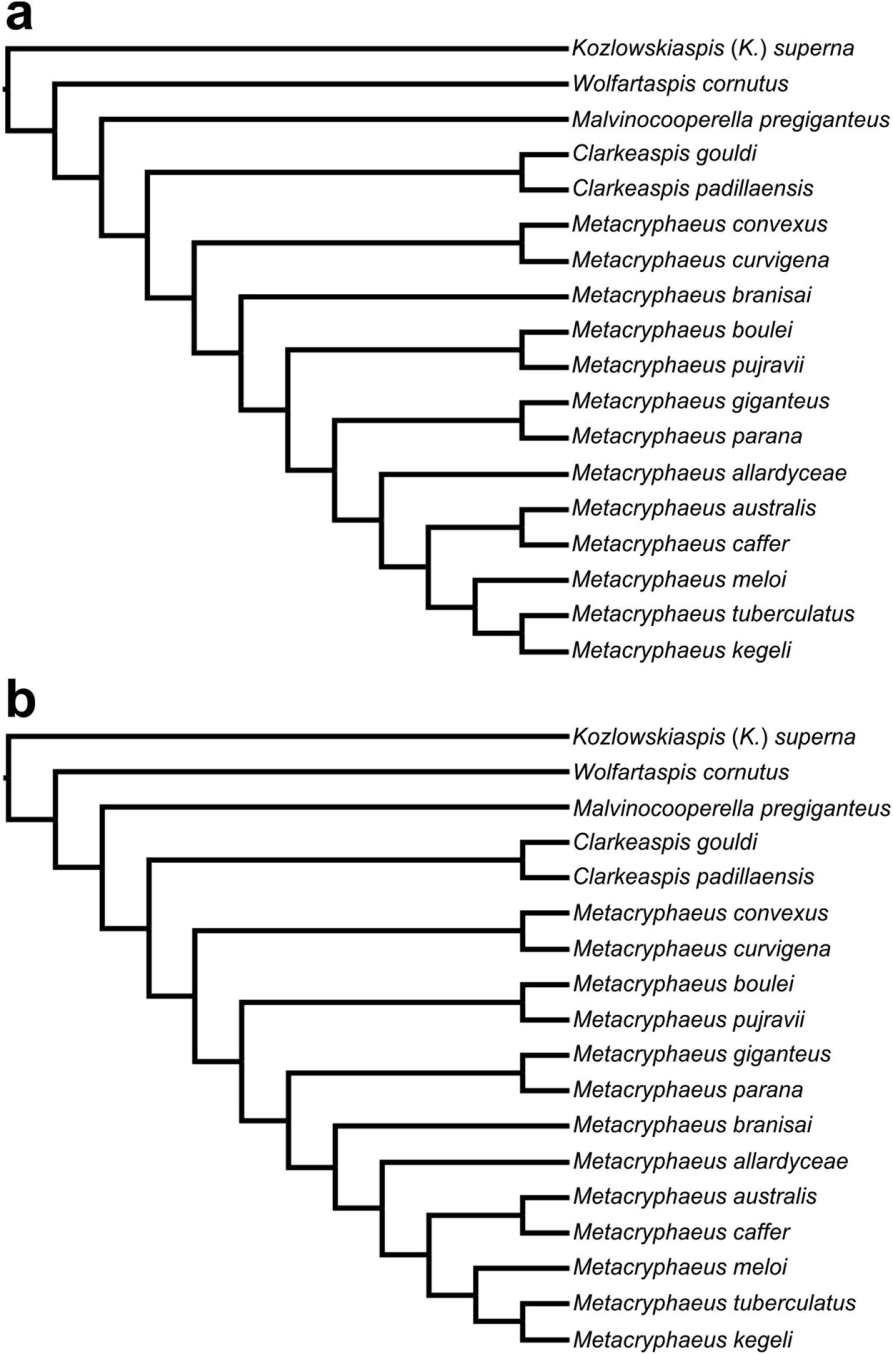
(**a**,**b**) Two most parsimonious trees (132 long and consistency index of 0.41) calculated in the present phylogenetic analysis.

**Figure 5:**
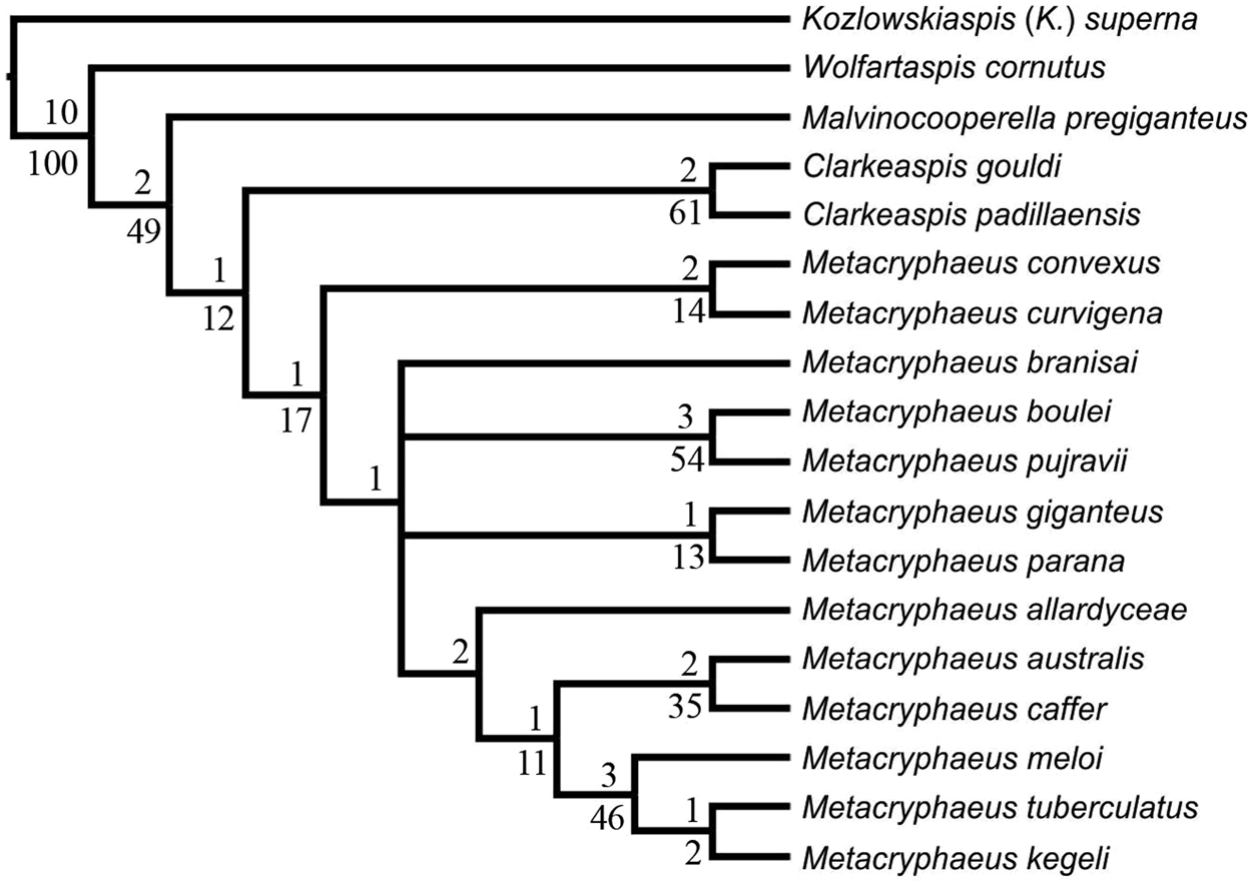
Strict consensus of the two MPTs with bootstrap values (using 1000 replicates; below) and Bremer support (above) indicated for each node.

The parsimony analysis resulted in two MPTs of 132 steps (consistency index = 0.41 and retention index = 0,52; Fig. 4). The only topological difference between these two trees is the placement of *Metacryphaeus branisai*. The strict consensus is presented in Fig. 5, along with bootstrap probabilities and Bremer decay indices for each node.

*Plesiomalvinella boulei* and *P. pujravii* were found deeply nested within a clade of *Metacryphaeus* species. Accordingly, those two species are here referred to that genus, as previously proposed by Wolfart^13^. *Metacryphaeus* (including *M. boulei* and *M. pujravii*) is here supported by two synapomorphies: frontal lobe projecting beyond the cephalic anterior border in dorsal view (character 4) and uniformly divergent axial furrows from SO to the cephalic margin (character 19).

In contrast to Lieberman^5^, *Clarkeaspis gouldi* (Lieberman, 1993) and *C. padillaensis* (Lieberman, 1993) were grouped into a clade supported by four synapomorphies (Figs 4 and 5): cephalic anterior border (cranidial) extended and pointed (characters 2 and 3); pentagonal glabella (character 6); 60 to 70% ratio between the basal glabellar width and the glabellar length (character 9). *Clarkeaspis* is here placed closer to *Metacryphaeus* (as its sister group) than in Lieberman^5^. The *Metacryphaeus* + *Clarkeaspis* clade is supported by a single synapomorphy (character 9, 0 → 1) and shows low bootstrap support (Fig. 5). The placement of *Malvinocooperella pregiganteus* (Lieberman, 1993) and *Wolfartaspis cornutus* (Wolfart 1968) as successive outgroups of the *Metacryphaeus* + *Clarkeaspis* (Figs 4 and 5), also differs from the arrangement seen in Lieberman^5^.

The present analysis recovered the clades formed by *Metacryphaeus giganteus* + *M. parana* (Figs 4 and 5) and *M. boulei* + *M. pujravii* (Figs 4 and 5), previously recognized by Lieberman^5^. Synapomorphies of the *M. giganteus* + *M. parana* clade are: 60 to 70% ratio between the basal glabellar width and the glabellar length (character 9), convergently acquired in *Clarkeaspis*; slender genal spine (character 36); dorsoventral height of the pygidium gradually decreasing posteriorly (character 39); 0.65 to 0.80 ratio between the maximum pygidial axial width and the maximum pygidial axial length (character 42). The *M. boulei* + *M. pujravii* clade is supported by six synapomorphies which are related to the presence of two symmetrical rows of sagittal spines on the posterior part of the glabella (character 15), the presence of one or two spines on L1 and L2 (characters 17 and 18), 0.15 and 0.25 ratio between the distance of posterior margin of the eyes to the axial furrow and the maximum glabellar width (character 25), the presence of four or five spines on the thoracic axial rings (character 37), and the prosopon covered by spines (character 48).

Our study also recovered new hypotheses for the relationships of *Metacryphaeus*, including a clade formed by *M. allardyceae*, *M. caffer*, *M. australis*, *M. meloi*, *M. kegeli*, and *M. tuberculatus*. This is supported by four synapomorphies related to the shape and extension of the (cranidial) cephalic anterior border (characters 2 and 3), the ratio between the sagittal length of L1 and the glabellar sagittal length (character 14), and the incision of the occipital furrow medially (character 29). The clade including *M. caffer*, *M. australis*, *M. meloi*, *M. kegeli*, and *M. tuberculatus* is supported by four synapomorphies (Fig. 4a): glabella posteriorly elevated and declined anteriorly to S3 (character 8); 65 to 75° α angle (character 22); rounded pygidial terminus (character 45); no spine on the pygidial terminus (character 46). Also, the clade formed by *M. tuberculatus*, *M. meloi*, and *M. kegeli* is supported by four synapomorphies related to L2 and L3 that do not merge distally (character 13), 55 to 64° β angles (character 23), the connection of S2 and the axial furrow (character 24), and the lack of connection between the anterior margin of the eyes and the axial furrow (character 26) (Fig. 5). Two synapomorphies support the *M. caffer* plus *M. australis* clade: characters 9 (reverted to the plesiomorphic condition) and 41, which are respectively related to a ratio greater than 80% between the basal glabellar width and the glabellar length, and to 0.25 to 0.35 ratios between the maximum pygidial axial width and the maximum pygidial width.

The clade that includes all *Metacryphaeus* except for *M. convexus*, *M. curvigena*, and *M. branisai* (Fig. 4a) is supported by three synapomorphies related to a 0.15 to 0.25 ratio between the distance from the posterior margin of the eyes to the axial furrow and the maximum glabellar width (character 25), occipital furrow weakly incised medially (character 29), and 130 to 160° γ angle (character 34). Three synapomorphies support the group formed by *M. giganteus*, *M. parana*, *M. allardyceae*, *M. australis*, *M. caffer*, *M. meloi*, *M. kegeli*, and *M. tuberculatus* (Fig. 4a): 0.25 to 0.34 ratio between sagittal length of L1 glabellar lobe and glabellar sagittal length (character 14), 0.3 to 0.4 ratio between the maximum exsagittal eyes length and the glabellar sagittal length (character 27), 0.60 to 0.80 ratio between maximal sagittal pygidial length and maximal transverse pygidial width (character 40). The position of *Metacryphaeus branisai* is variable in the two MPTs (Fig. 4), probably because its pygidium is unknown, implying the non-codification for characters 38 to 47.

In the phylogeny modelled by Lieberman^5^, *Metacryphaeus convexus* and *M. curvigena* are not considered sister taxa to all other *Metacryphaeus*. Instead, *M. curvigena* is considered the sister taxon to *M. branisai* and *M. convexus* the sister taxon to both (Fig. 1a). In our analysis, the clade formed by *M. convexus* and *M. curvigena* is supported by five synapomorphies: inclination of 10–20° of S3 in relation to SO (character 12); L2 and L3 not merged distally (character 13); cephalic axial furrows deep and broad (characters 20 and 21); evident connection between S2 and the axial furrow. Likewise, the affinities of *M. meloi* and *M. kegeli* are supported by four synapomorphies. This is interesting because these species are endemic to the Parnaíba Basin (Brazil), as is their sister-taxon *M. tuberculatus*, the only other species of the genus known to that basin.

### Palaeobiogeography

Likelihood Ratio Test supports DEC M2 (*w* and *j* set as free parameters) as the best-fit model to our data (Table 1). The palaeobiogeographic reconstructions differ only slightly for the two MPTs, so we focus the discussion on the first MPT. The summary of biogeographic stochastic mapping (BSM) counts (Table 2) shows a predominance of dispersals among range change events (33.6% of total events) and, among those, founder events (19.6%) are slightly more frequent than anagenetic dispersals (14.1%). Vicariance was very uncommon according to our model, accounting only for 3.9% of the events (Table 2). Most dispersals occurred from Bolivia and Peru (A) to other areas, more frequently to the Paraná (B) and Parnaíba (E) basins (Table 3).

**Table 1:** Pairwise comparison of the results of the ancestral area reconstructions of nested DEC models on tree 1.

Alternative Model	LnL	DF	Null Model	LnL	DF	Likelihood Ratio Test *p*
DEC + w	−32.41	3	DEC	−32.98	2	0.29
DEC + w + j	−29.87	4	DEC + w	−32.41	3	0.024

Abbreviations: LnL, log-likelihood values; DF, degrees of freedom.

**Table 2:** Summary of BSM (Biogeographic Stochastic Mapping) counts based on DEC M2 model showing the mean, standard deviations (SD), and percentage of different types of biogeographic events.

Type	Mean (SD)	%
range contractions (e)	0 (0)	0.0%
range expansion (d)	2.78 (0.60)	14.1%
founder events (j)	3.87 (1.33)	19.6%
all dispersals	6.65 (0.97)	33.6%
sympatry (y)	11.01 (0.81)	55.7%
subset speciation (s)	1.34 (0.93)	6.7%
vicariance (v)	0.78 (0.60)	3.9%

**Table 3:** Counts (and standard deviations in parentheses) of dispersal events averaged across 100 biogeographics stochastic mappings based on the biogeographic history of *Metacrypheus* according to DEC M2 model.

	A	B	C	D	E
A	0 (0)	2.42 (0.64)	0.11 (0.31)	0.69 (0.46)	1.39 (0.55)
B	0.53 (0.69)	0 (0)	0.87 (0.34)	0.31 (0.46)	0.03 (0.17)
C	0.02 (0.14)	0.16 (0.37)	0 (0)	0 (0)	0 (0)
D	0 (0)	0.10 (0.30)	0.02 (0.14)	0 (0)	0 (0)
E	0 (0)	0 (0)	0 (0)	0 (0)	0 (0)

(A) Bolivia and Peru, (B) Paraná Basin, (C) South Africa, (D) Falkland Islands, and (E) Parnaíba Basin.

**Figure 6:**
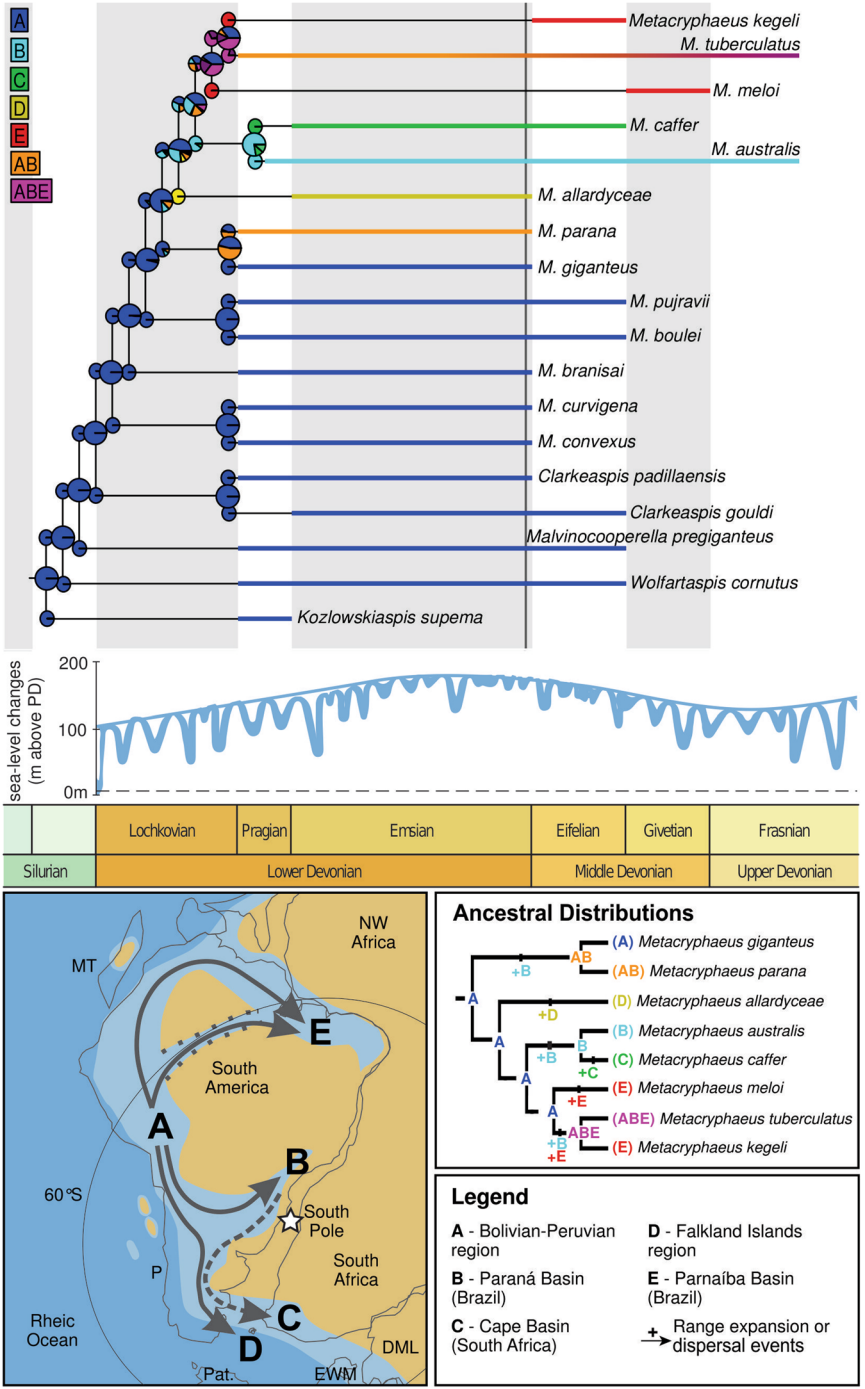
Ancestral area reconstructions based on DEC M2 model on the tree 1 (top), sea-level changes curves from Lochkovian to Frasnian (middle) based on Haq & Schutter^55^, and Lower Devonian palaeomap of Southern Gondwana (bottom) modified from Torsvik & Cocks^56^. Arrows on the palaeomap indicate inferred Lochkovian (full arrow) and Pragian (dashed arrow) dispersal routes for *Metacryphaeus* taxa. Additional abbreviations: DML, Dronning Maud Land, Antarctica; EWM, Ellsworth-Whitmore Mountains, Antarctica; MT, Mexican terranes; P, Precordillera Terrane, Argentina; Pat., Patagonia.

All three models estimate a 100% probability for Bolivia and Peru (A) as the ancestral area for the *Metacryphaeus* clade, as well as for most of its internal clades (Fig. 6; Supplementary Supple 3). The earliest *Metacryphaeus* records in this area are from the early Pragian^4,5^, but three range changes were estimated to have occurred earlier, during the late Lochkovian (Fig. 6): 1- the ancestor of *M. parana* and *M. giganteus* expanded its occurrence to encompass the Paraná Basin (B), with the former species maintaining this broader distribution and the latter restricted to B (subset sympatry) - in an alternative scenario, the ancestor of this clade is present only in Bolivia and Peru (A), with *M. parana* expanding its range to also the Paraná Basin (B); 2- *M. allardyceae* dispersed to the Falklands area (D); 3- the ancestor of *M. australis* and *M. caffer* dispersed to the Paraná Basin (B). During the early Pragian, *M. caffer* dispersed from the Paraná Basin to South Africa (C). It is interesting to note that those dispersal and expansion events likely occurred before the transgressive events on western Gondwana^14–17^ dated between the late Pragian and the early Emsian (Fig. 6). Those areas (A, B, C, D) were eventually connected by transgressive-regressive cycles (Fig. 6), which promoted the faunal similarity observed among the Malvinokaffric fauna of the Early Devonian^15,18^.

The last common ancestor of *Metacryphaeus meloi*, *M. kegeli*, and *M. tuberculatus*, and the node including only the latter two taxa were reconstructed with two almost equal probable ranges, either restricted to Bolivia and Peru (A) or a joint distribution (Fig. 6) also including the Parnaíba and Paraná basins (ABE). These different ancestral range reconstructions imply distinct processes of range changes, respectively: 1 - successive dispersals from Bolivia and Peru to the other areas (for an ancestral with distribution restricted to A), 2 - distribution expansions inferred as founder events (for an ancestral widely distributed in ABE). Nevertheless, in all cases *M. meloi* and *M. kegeli* became restricted to the Parnaíba Basin (E), whereas *M. tuberculatus* maintained (or reached) a widespread distribution (ABE), even though its earliest records, dated as late Eifelian and early Givetian, do not include the Parnaíba Basin^4–6,11,12^. Alternatively, but with lower statistical support, the ancestral range reconstruction hypothesized for the clades *M. meloi* + (*M. tuberculatus* + *M. kegeli*) and *M. tuberculatus* + *M. kegeli* could be AB, encompassing only their older records. This would imply expansion events towards the Parnaíba Basin (E) after the arrival of ancestors in the Paraná Basin (B).

The arrival of *Metacryphaeus* in the Parnaíba Basin may have occurred via two alternative routes (Fig. 6). A northern route (surrounding the northern margin of the South American continent) would impose no continental (landmass) barriers, but there would be climatic barriers related to the warmer waters the animals would need to overcome, as the Malvinokaffric Realm marks cooler areas. Also, faunas of this age on the northern margin of South-America belong to other realms, which lack *Metacryphaeus*. On the other hand, a route through the Amazon Basin (Fig. 6) would have presented no climatic or faunal barriers (*cf*.^15,18,19^). Even a continental barrier might not have been in place, as there were transgression events possibly connecting that basin to Bolivia and Peru. The lack of fossils of this age in the Amazon Basin, which could confirm such a dispersal route, is related to the depositional gap present in the upper Lochkovian and lower Emsian of the basin (*cf*.^20–25^). This absence of Lochkovian–lower Emsian rocks is also observed in the Parnaíba Basin^20,21,24^, which hinders palaeobiogeographical inferences related to the presence/absence of *Metacryphaeus* in the Lower Devonian of this basin.

Other trilobite genera also have a broad Gondwanan distribution during the Devonian, *e.g*. the calmoniid *Eldredgeia*, with occurrences in the Bolivia, Brazil (Amazon and Parnaíba basins), and South Africa, and the homalonotid *Burmeisteria*, with records in the Brazil (Amazon, Parnaíba, and Paraná basins), Falkland Islands, South Africa, and Ghana^1,15,19,26^. Furthermore, the distribution of the brachiopods *Tropidoleptus carinatus* (Conrad, 1839) and *Australocoelia palmata* (Mooris & Sharpe, 1846), and the crinoids *Exaesiodiscus* Moore & Jeffords, 1968, *Laudonomphalus* Moore & Jeffords, 1968, *Monstrocrinus* Schmidt, 1941, and *Marettocrinus* Le Menn^15,27–34^, also reinforce that connections between the Bolivian-Peruvian region and the Amazon, Parnaíba, and Paraná basins were recurrent by the Middle Devonian (e.g.^15,26^). However, the dispersal and range expansion events highlighted in our biogeographic analyses (except that related to *M. caffer* dispersal from the Paraná Basin to South Africa) occurred during the late Lochkovian (Fig. 6). As such, our data suggest an earlier connection between all those Gondwanan regions, allowing *Metacryphaeus* trilobites to expand into the Paraná and Parnaíba basins via southeastern and northern/northeastern routes, respectively (Fig. 6). Another interesting fact is the diversification of *Metacryphaeus* in South America occurring earlier than its dispersal to South Africa (where it is represented by *M. caffer*). This was temporally the latest dispersal of the genus, taking place during the Pragian, and a separate event from the dispersal of *M. allardyceae* in the same direction (to the Falkland Islands), which occurred earlier.

## Methods

### Phylogenetic analysis

The phylogenetic analysis conducted here was based on the phylogeny of Lieberman^5^, with extra characters and species added to the data matrix. The added species were *Metacryphaeus australis*, *M. caffer*, *M. kegeli*, *M. meloi*, and *M. allardyceae*, as to encompass all valid species of the genus. Other ingroup taxa were defined according to the phylogenetic hypothesis of Lieberman^5^ consisting of *Plesiomalvinella boulei*, *P. pujravii*, *Wolfartaspis cornutus*, *Malvinocooperella pregiganteus*, *Clarkeaspis gouldi*, and *C. padillaensis*. Also, according to Lieberman^5^, *Kozlowskiaspis* (*K*.) *superna* Braniša & Vaněk, 1973 was used to root of the phylogenetic trees.

**Figure 7:**
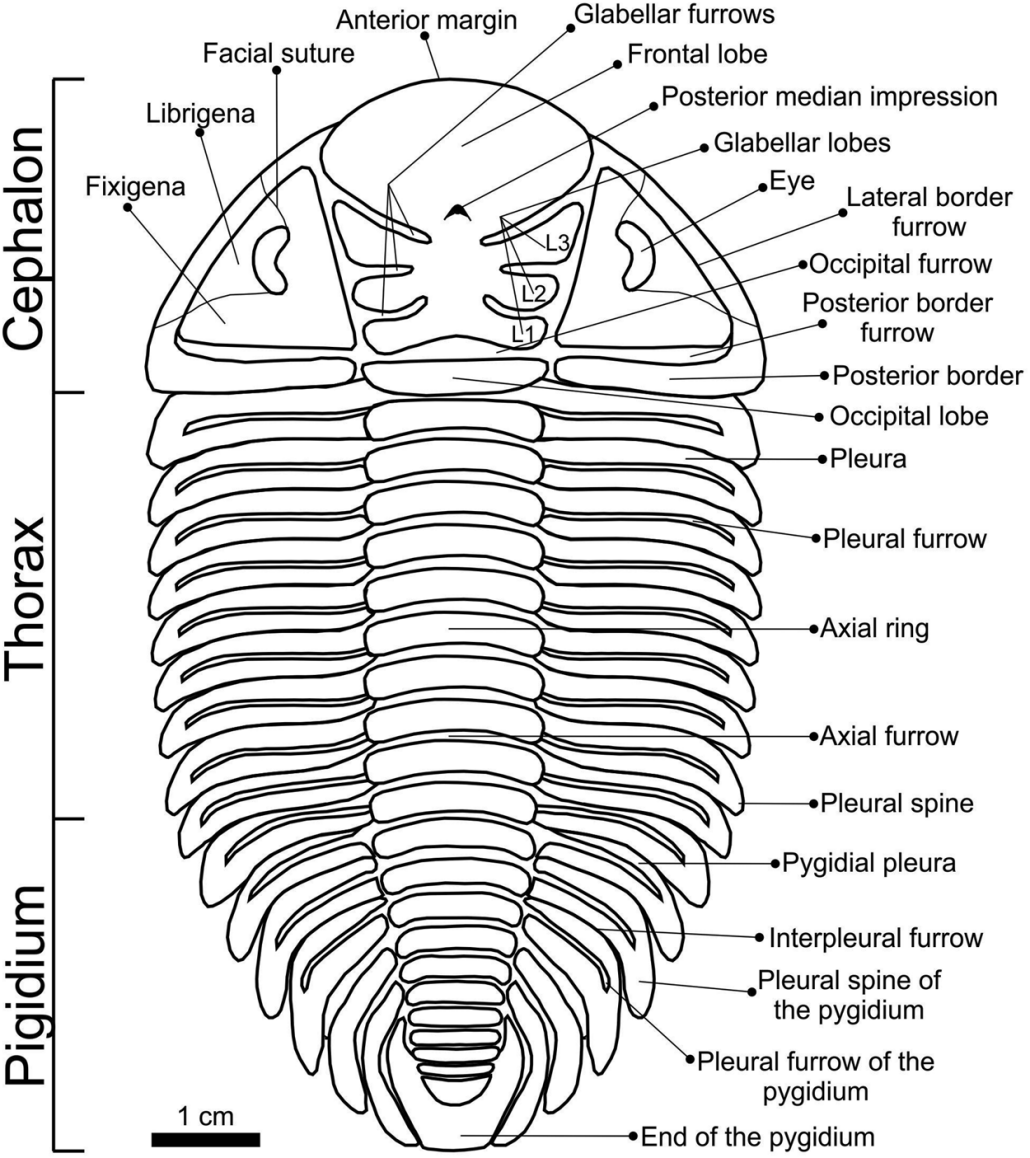
Schematic drawing showing the major exoskeleton elements of the dorsal surface of *Metacryphaeus*.

**Figure 8:**
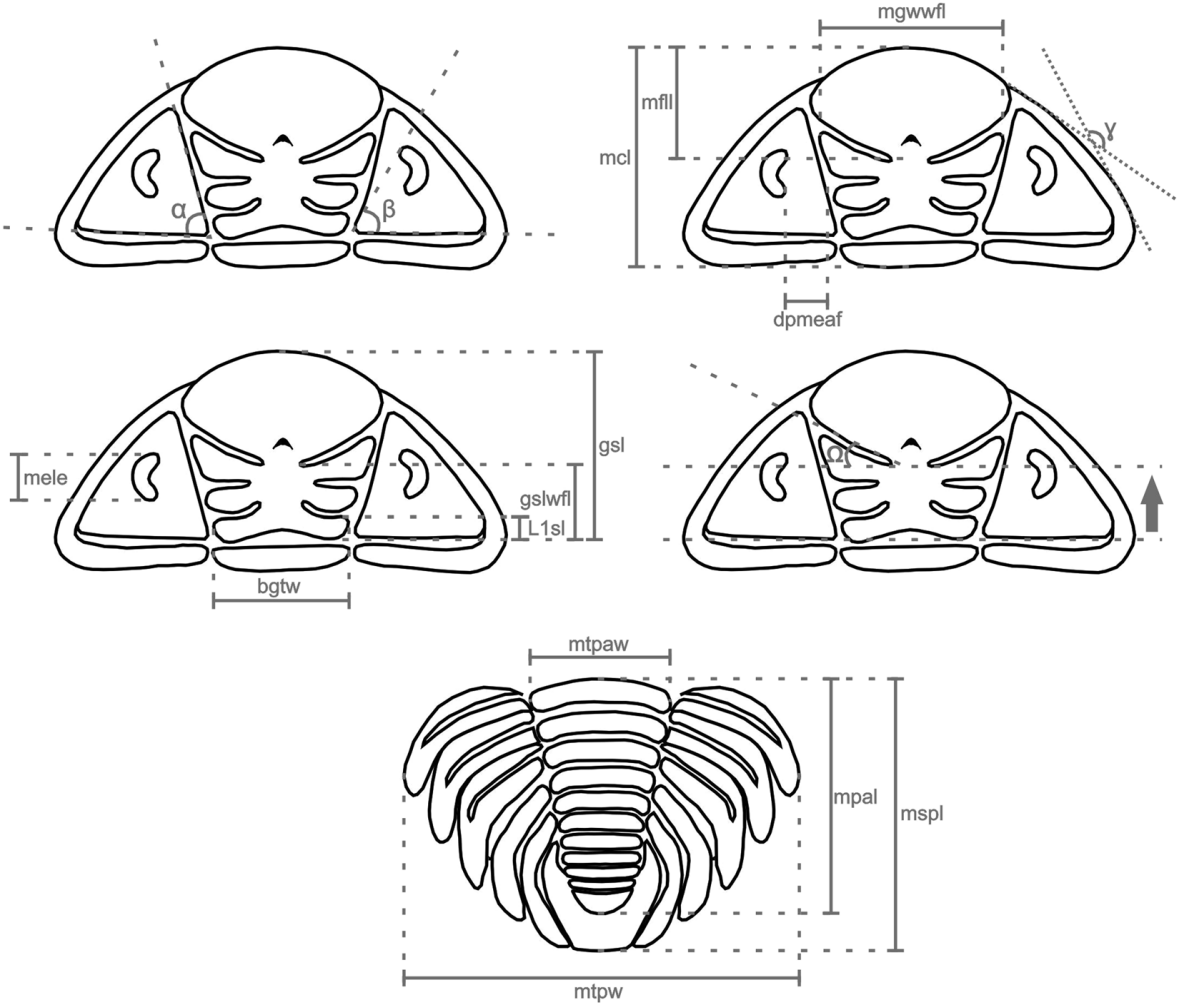
Measurements used: mgwwfl = maximum glabelar width without consider the frontal lobe; mfll = maximum frontal lobe length; mcl = maximum cephalic length; dpmeaf = distance of posterior margin of the eyes to the axial furrow; mele = maximum exsagittal length of the eyes; bgtw = basal glabellar transverse width; gsl = glabellar sagittal length; gslwfl = glabellar sagittal length without consider the frontal lobe; L1sl = sagittal length of L1 glabellar lobe; mtpaw = maximum transverse pygidial axis width; mtpw = transverse maximum pygidial width; mspl = sagittal maximum pygidial length; mpal = maximum pygidial axis length; α = angle between the axial furrow and the furrow of cephalic posterior border; β = angle between the cephalic posterior border furrow and a line traced from the posterior margin of the axial furrow to the anterior margin of the eyes; γ = angle between a straight line traced adjacent to the lateral genae (from the contact with the cephalic posterior furrow) and a line traced from the anterior part of the genae (from the contact of the axial furrow) in direction to the medial-posterior part of the genae; Ω = S3 inclination in relation to SO.

Among the 48 characters employed here (see Appendix 1), 33 were taken or modified from Lieberman^5^ and 15 are new (characters 7, 9, 12, 13, 14, 22, 23, 24, 25, 26, 27, 34, 40, 41, and 42), although based on characters used in phylogenetic analyses of other trilobite groups (*e.g*.^35–42^). The morphological elements of the exoskeleton are shown in Fig. 7 and all morphological relations/angles used in the 15 newly proposed characters were measured as indicated in Fig. 8.

Among the characters taken from Lieberman^5^, some scores were changed for some taxa based on our own interpretations. This is the case for characters 5 (changed from 0 to 1 in *Malvinocooperella pregiganteus*, *Metacryphaeus giganteus*, and *Me. branisai*), 18 (changed from 0 to 1 in *Me. branisai*), and 19 (changed from 0 to 1 in *Me. giganteus*). Other characters from Lieberman^5^, *e.g*. characters 9, 10, 12, 13, 19, 23, 24, and 34, were not used here because they either have too much variation between individuals of the same species or can be easily affected by taphonomic deformation. Some characters from Lieberman^5^ were split into two or more characters, as in case of characters 2 and 3 (=character 1 of Lieberman^5^), 20 and 21 (=character 18 of Lieberman^5^), 30 and 31 (=character 25 of Lieberman^5^), 35 and 36 (=character 29 of Lieberman^5^), and 45, 46, and 47 (=character 36 of Lieberman^5^), following a contingential approach^43^.

Characters 1 to 36 are related to the cephalon, character 37 to the thorax, 38 to 47 to the pygidium, and 48 to the prosopon (Appendix 1 and 2). All characters are related to the dorsal surface of the exoskeleton and were treated as ordered. The data matrix was analyzed in search of the Most Parsimonious Trees (MPTs) using the software TNT version 1.1^44^. A heuristic search was conducted with 1,000 replicates, random addition of taxa (random seed 0), *Tree Bisection and Reconnection* (TBR) as branch swapping algorithm, and “hold” of 10 trees per replica. The recovered MPTs were summarized in a strict consensus tree. Bremer^45^ decay indices and bootstrap proportions^46^ were calculated using scripts incorporated in TNT. The data matrix was compiled in NEXUS format using the software Mesquite version 3.03 (702) and the tree images were generated with the software FigTree version 1.4.2.

### Palaeobiogeographical analysis

We conducted palaeobiogeographic analyses to explore the distribution dynamic and biogeographical events that affected *Metacryphaeus* distribution through time in five areas pre-defined based on the known occurrences genus: Bolivia and Peru (A); Paraná Basin, Brazil (B); South Africa (C); Falkland Islands (D); and Parnaíba Basin, Brazil (E). Bolivia and Peru were treated as a single area due to their geographical proximity, strong palaeontological association, and co-occurrence of endemic species^2,47^. Only fossil taxa with accurate occurrence data and taxonomic identification were included. For this reason, taxa with doubtful assignation (*cf*., *aff*.) were not considered in our analyses (*e.g*.^48^).

Ancestral area reconstructions were conducted using *R* (R Development Core Team 2013) package *BioGeoBEARS*^49^, which allows comparing the likelihood of our data given distinct models, choosing that with better fit^50^. We tested three nested models based on the LAGRANGE Dispersal-Extinction-Cladogenesis (DEC) model^51,52^: M0 contains the default parameters of the DEC models^49^; M1 has the addition of the free parameter *w*; and M2 has the addition of the free parameters *w* and *j*. The free parameter *w* is a multiplier of the dispersal matrices and when set to 1 (*e.g*. in M0) the probabilities of dispersal events are based solely on the dispersal matrices and equal across all events^53^. The founder-event parameter *j* (included only in M2 and set to 0 in M0 and M1) allows range changes to areas distinct to that of the ancestor during a cladogenetic event^49^. We employed the Likelihood Ratio Test (LRT) to select the best model.

We used time-calibrated versions of the two MPTs, dividing them into two time slices, Silurian to Lower Devonian (430–395 Ma) and Middle to Upper Devonian (395–382 Ma). Based on that, we conducted a time-stratified analyses using time-specific dispersal multiplier and area matrices (see Supplementary supple 2 and supple 3). This allowed changing the distances and probabilities between the areas along these periods, simulating the continental transformations.

We also conducted a biogeographic stochastic mapping (BSM) on *BioGeoBEARS*^54^ to estimate the number and type of biogeographical events. We conducted the BSM only for the first MPT, as the ancestral area reconstruction of both MPTs differ only slightly, and employed the parameters of the best-fit model of the ancestral area reconstruction^53^. The mean and standard deviation of event counts of 100 BSMs were used to estimate the frequencies of range change between the considered areas and of each kind of biogeographic event.

## Conclusions

This work provides new phylogenetic hypotheses for the relationships of all species within the genus *Metacryphaeus*, including the identification of the clades composed of (1) *M. caffer* and *M. australis*, (2) *M. tuberculatus*, *M. meloi*, and *M. kegeli*, (3) *M. tuberculatus* and *M. kegeli*, (4) *M. curvigena* and *M. convexus*, the latter two as sites clades. The position of *M. branisai* varied in the two recovered MPTs, probably due to the unknown pygidium for this specie. As *Plesiomalvinella pujravii* and *P. boulei* were positioned within the *Metacryphaeus* clade, these species were reinserted in that genus, as originally suggested by Wolfart^13^. Finally, the genus *Clarkeaspis* represents the immediate outgroup to *Metacryphaeus*.

The results of the palaeobiogeographic analyses with DEC models reinforce the interpretations of Lieberman^5^ and Abe & Lieberman^9^ that *Metacryphaeus* originated in the Lower Devonian of Bolivia and Peru, where they are represented by a higher taxonomic diversity. The radiation of *Metacryphaeus* to other Gondwanan regions probably occurred during the transgressive events in the Lochkovian–Pragian. In the Lochkovian, dispersals would have occurred to the Paraná Basin, in Brazil (*M. parana, M. australis*, *M. tuberculatus*), as well as to the Falklands area (*M. allardyceae*) and the Parnaíba Basin (*M. meloi, M. kegeli*, *M. tuberculatus*). Pragian dispersal events were reconstructed only towards South Africa (*M. caffer*).

The ancestral area reconstructions for *Metacryphaeus* show dispersal events occurring earlier than expected, *i.e*. during the Early Devonian, even though the faunal similarities of Bolivia and Peru with the Parnaíba and Amazon basins are more prominent in the Middle Devonian, with the sharing of brachiopod (*Tropidoleptus* and *Australocoelia*), crinoid (*Exaesiodiscus*, *Laudonomphalus*, *Monstrocrinus*, and *Marettocrinus*), and other trilobite (*Eldredgeia* and *Burmeisteria*) taxa. The results presented here indicate that these areas were also somehow connected during the beginning of the Devonian, as to allow the dispersal of *Metacryphaeus*.

## Electronic supplementary material

Supplementary Materials and Appendices

## Data Availability

The datasets analyzed during the current study are available in: https://figshare.com/s/6b42cf2d4d0cadde7e11.

## References

[1] Clarke, J. M. Fósseis devonianos do Paran*á* (Monografia, vol. 1) 1–353 (Serviço Geológico e Mineralógico do Brasil, 1913).

[2] Eldredge, N. & Ormiston, A. R. In: Gray, J., Boucot, A. J., editors. *Historical biogeography, plate tectonics, and the changing environmen*t. Biogeography of Silurian and Devonian trilobites of the Malvinokaffric realm. 147–167 (1979).

[3] Cooper MR (1982). A revision of the Devonian (Emsian-Eifelian) Trilobita from the Bokkeveld Group of South Africa. Annals of the South African Museum.

[4] Lieberman BS, Edgecombe GD, Eldredge N (1991). Systematics and biogeography of the “Malvinella group”, Calmoniidae (Trilobita, Devonian). Journal of Paleontology.

[5] Lieberman BS (1993). Systematics and biogeography of the “*Metacryphaeus* Group”, Calmoniidae (Trilobita, Devonian), with comments on adaptative radiations and the geological history of the Malvinokaffric Realm. Journal of Paleontology.

[6] Carvalho MGP, Edgecombe GD, Lieberman BS (1997). Devonian Calmoniid trilobites from the Parnaíba Basin, Piauí State, Brazil. American Museum Novitates.

[7] Ghilardi RP, Simões MG (2007). History and development of trilobite research in Brazil. New York State Museum Bulletin.

[8] Velazco YP (2012). Fósiles del paleozoico de la colección Antonio Raimondi conservados en el Museo de Historia Natural – Universidad Nacional Mayor de San Marcos, Lima-Perú, 1° parte. Revista del Instituto de Investigación de la Facultad de Ingeniería Geológica, Minera, Metalurgica y Geográfica.

[9] Abe FR, Lieberman BS (2009). The nature of evolutionary radiations: a case study involving Devonian trilobites. Evolutionary Biology.

[10] Abe FR, Lieberman BS (2012). Quantifying morphological change during an evolutionary radiation of Devonian trilobites. Paleobiology.

[11] Meira F, van E, Carbonaro FA, Ghilardi RP, de M. Leme J (2016). The *“Metacryphaeus tuberculatus* group” (Trilobita, Calmoniidae) from the Devonian of the Parnaíba Basin, Brazil. Ameghiniana.

[12] Carbonaro FA (2016). *Metacryphaeus tuberculatus* and *Metacryphaeus australis* (Trilobita, Phacopida) from the Devonian of the Paraná Basin: Taxonomy and Palaeobiogeography. Ameghiniana.

[13] Wolfart, R. In: Wolfart, R. & Voges, A., editors. Beihefte zum Geologischen Jahrbuch. Vol. 74. *Beitriige zur Kenntnis des Devons von Bolivien*. Die Trilobiten aus dem Devon Boliviens und ihre Bedeutung fir stratigraphie und tiergeographie. 5–201 (1968).

[14] Boucot AJ, Gray J (1983). A Paleozoic Pangaea. Science.

[15] Melo, J. H. G. In: McMillan, N. J., Embry, A. F. & Glass, D. J., editors. Canadian Society of Petroleum Geologists. Memoir 14. *Devonian of the World*. The Malvinokaffric Realm in the Devonian of Brazil. 669–703 (1988).

[16] Assine ML, Perinotto JAJ, Fulfaro VJ, Petri S (1998). Progradação deltáica Tibagi no Devoniano Médio da Bacia do Paraná. Revista Brasileira de Geociências.

[17] Bosetti EP, Grahn Y, Horodyski RS, Mendlowics Mauller P, Breuer P (2012). The first recorded decline of the Malvinokaffric Devonian fauna in the Paraná Basin (southern Brazil) and its cause; taphonomic and fossil evidences. Journal of South American Earth Sciences.

[18] Boucot AJ (1971). Malvinokaffric Devonian marine community distribution and implications for Gondwana. Anais da Academia Brasileira de Ciências.

[19] Carvalho MGP, Ponciano LCM (2015). The Devonian trilobites of Brazil: A summary. Journal of South American Earth Sciences.

[20] Berry WB, Boucot AJ (1973). Glacio-eustatic control of Late Ordovician–Early Silurian platform sedimentation and faunal changes. Geological Society of America Bulletin.

[21] Caputo, M. V. & Lima, E. C. In: Sociedade Brasileira de Geologia. Vol. 8. *Anais 33° Congresso Brasileiro de Geologia, Rio de Janeiro*. Estratigrafia, idade e correlação do Grupo Serra Grande–Bacia do Parnaíba. 740–753 (1984).

[22] Melo JHG, Loboziak S (2003). Devonian–Early Carboniferous miospore biostratigraphy of the Amazon Basin, Northern Brazil. Review of Palaeobotany and Palynology.

[23] Grahn Y (2005). Silurian and Lower Devonian chitinozoan taxonomy and biostratigraphy of the Trombetas Group, Amazonas Basin, northern Brazil. Bulletin of Geosciences.

[24] Vaz PT, Rezende NGAM, Wanderley Filho JR, Travassos WS (2007). Bacia do Parnaíba. Boletim de Geociências da PETROBRAS.

[25] Cunha PRC, Melo JHG, Silva OB (2007). Bacia do Amazonas. Boletim de Geociências da PETROBRAS.

[26] Carvalho MGP, Fonseca VMM (2007). The Trilobite “*Dalmanites*” *Maecurua* (Middle Devonian, Amazon Basin, Brazil) and the New Genus *Amazonaspis* (Synphoriidae). American Museum Novitates.

[27] Isaacson PE, Perry DG (1977). Biogeography and morphological conservatism of *Tropidoleptus* (Brachiopoda, Orthida) during the Devonian. Journal of Paleontology.

[28] Melo, J. H. G. *A Província Malvinocáfrica do Devoniano do Brasil – Estado atual dos conhecimentos*. Master Thesis, Universidade Federal do Rio de Janeiro (1985).

[29] Fonseca, V. M. M. & Melo, J. H. G. In: Sociedade Brasileira de Paleontologia. 10 *Congresso Brasileiro de Paleontologia*. Ocorrência de *Tropidoleptus carinatus* (Conrad) (Brachiopoda, Orthida) na Formação Pimenteira, e sua importância paleobiogeográfica. 505–537 (1987).

[30] Petri, S. & Fulfaro, V. J. Geologia do Brasil (2ª ed., ed. Queiroz, T. A.) 1–631 (Universidade de São Paulo, 1988).

[31] Assine MLO (2001). Ciclo Devoniano na Bacia do Paraná e correlação com outras bacias Gondwânicas. *Ciência, Técnica, Petróleo*. Seção Exploração de Petróleo.

[32] Gama, J. M. Jr. *Braquiópodes da Formação Pimenteiras (Devoniano Médio/Superior), na região sudoesta da Bacia do Parnaíba, município de Palmas, estado do Tocantins, Brasil*. Master Thesis, Universidade de Brasília (2008).

[33] Scheffler, S. M. *Crinóides e blastóides do Devoniano brasileiro*. PhD Thesis, Universidade Federal do Rio de Janeiro (2010).

[34] Scheffler SM, Silva SD, Gama JM, Fonseca VMM, Fernandes ACS (2011). Middle Devonian crinoids from the Parnaiba Basin (Pimenteira Formation, Tocantins State, Brazil). Journal of Paleontology.

[35] Brezinski DK (2008). Phylogenetics, systematics, paleoecology, and evolution of the trilobite genera Paladin and Kaskia from the United States. Journal of Paleontology.

[36] Lee SB, Lee DC, Choi DK (2008). Cambrian–Ordovician trilobite family Missisquoiidae Hupé, 1955: systematic revision and palaeogeographical considerations based on cladistic analysis. Palaeogeography, Palaeoclimatology, Palaeoecology.

[37] Congreve CR, Lieberman BS (2008). Phylogenetic and biogeographic analysis of Ordovician homalonotid trilobites. The Open Paleontology Journal.

[38] Congreve CR, Lieberman BS (2010). Phylogenetic and biogeographic analysis of deiphonine trilobites. Journal of Paleontology.

[39] Congreve CR, Lieberman BS (2011). Phylogenetic and biogeographic analysis of sphaerexochine trilobites. PloS one.

[40] Gapp IW, Lieberman BS, Pope MC, Dilliard KA (2011). New olenelline trilobites from the Northwest Territories, Canada, and the phylogenetic placement of Judomia absita Fritz, 1973. Zootaxa.

[41] Gapp IW, Congreve CR, Lieberman BS (2012). Unraveling the phylogenetic relationships of the Eccoptochilinae, an enigmatic array of Ordovician cheirurid trilobites. PloS one.

[42] Sundberg FA (2014). Phylogenetic analysis of the spiny oryctocephalids (Trilobita, Corynexochida?, Oryctocephalidae), Cambrian. Journal of Paleontology.

[43] Brazeau MD (2011). Problematic character coding methods in morphology and their effects. Biological Journal of the Linnean Society.

[44] Goloboff PA, Farris JS, Nixon KC (2008). TNT, a free program for phylogenetic analysis. Cladistics.

[45] Bremer K (1994). Branch support and tree stability. Cladistics.

[46] Felsenstein J (1985). Confidence limits on phylogenies: an approach using the bootstrap. Evolution.

[47] Isaacson, P. A. & Sablock, P. E. In: McMillan, N. J., Embry, A. F. & Glass, D. J., editors. Calgary: Canadian Society of Petroleum Geologists. *Devonian of the World*. Devonian system in Bolivia, Peru, and northern Chile. 719–728 (1988).

[48] Carvalho, M. G. P., Melo, J. H. G. & Quadros, L. P. In: Sociedade Brasileira de Paleontologia. *10 Congresso Brasileiro de Paleontologia*. Trilobitas devonianos do flanco noroeste da Bacia do Paraná. 545–565 (1987).

[49] Matzke, N. J. BioGeoBEARS: BioGeography with Bayesian (and likelihood) evolutionary analysis in R Scripts. *R package, version 0*.2, 1 (2013).

[50] Matzke NJ (2014). Model selection in historical biogeography reveals that founder-event speciation is a crucial process in island clades. Systematic Biology.

[51] Ree RH, Moore BR, Webb CO, Donoghue MJ (2005). A likelihood framework for inferring the evolution of geographic range on phylogenetic trees. Evolution.

[52] Ree RH, Smith SA (2008). Maximum likelihood inference of geographic range evolution by dispersal, local extinction, and cladogenesis. Systematic biology.

[53] Dupin J (2017). Bayesian estimation of the global biogeographical history of the Solanaceae. Journal of Biogeography.

[54] Matzke, N. J. Stochastic mapping under biogeographical models. PhyloWiki BioGeoBEARS website, http://phylo.wikidot.com/biogeobears#stochastic_mapping (2016).

[55] Haq BH, Schutter SR (2008). A chronology of Paleozoic sea-level changes. Science.

[56] Torsvik TH, Cocks LRM (2013). Gondwana from top to base in space and time. Gondwana Research.

