# Supplementary material for: Inferring ancestral range reconstruction based on trilobite records: a study-case on Metacryphaeus (Phacopida, Calmoniidae)
Source: Sci Rep. 2018 Oct 12;8:15179. doi: 10.1038/s41598-018-33517-5 (PMC6185901; doi:10.1038/s41598-018-33517-5)
Supplement: Supplementary file 1 — Supplementary Materials and Appendices [file 41598_2018_33517_MOESM1_ESM.pdf]

INFERRING ANCESTRAL RANGE RECONSTRUCTION ON TRILOBITE RECORDS: A STUDY-CASE BASED ON *METACRYPHAEUS* (PHACOPIDA, CALMONIIDAE)

FÁBIO AUGUSTO CARBONARO, MAX CARDOSO LANGER, SILVIO SHIGUEO NIHEI, GABRIEL DE SOUZA FERREIRA, RENATO PIRANI GHILARDI

All the supplementary information of this manuscript is present in the link below, except appendices 1 and 2 that are present in this pdf (pages 2-5).
https://figshare.com/s/6b42cf2d4d0cadde7e11.

Legend of the link content:

supple1 - Taxon-character matrix of morphological data with 18 taxa and 48 characters. The absence of data is indicated as "?".

supple2 - R scripts and input files.

supple3 - Results of the biogeographic (ancestral area reconstruction and biogeographic stochastic mapping) analyses.

Appendix 1. List of characters.

1. Medial portion of the cephalic anterior border: (0) not constricted; (1) constricted, so that the doublure contacts the cranidial border.

2. Forward extension of (cranidial) cephalic anterior border in dorsal view: (0) absent; (1) present.

3. Shape of the extension of (cranidial) cephalic anterior border: (0) broad and triangular; (1) long and pointed.

4. Frontal lobe projects beyond cephalic anterior border in dorsal view: (0) no; (1) yes.

5. Anteromedian bulge in frontal lobe in relation to the entire cephalon: (0) slight, little conspicuous; (1) detached.

6. Shape of anterior portion of frontal lobe: (0) pointed such that glabella is pentagonal; (1) rounded.

7. Maximum frontal lobe length (mfll): (0) 40 to 49% of the maximum cephalic length (mcl); (1) 50 to 60% of the maximum cephalic length (mcl).

8. Topography of the glabella (sag.) anterior to S3: (0) constant or declined anteriorly; (1) posteriorly elevated and declined anteriorly.

9. Ratio between the basal glabellar transverse width (bgtw) and the glabellar sagittal length (gsl): (0) $>$ 80%; (1) 70 – 80%; (2) 60 – 70%.

10. PMI (posterior median impression): (0) not visible; (1) varyingly discernible.

11. Incision of S3: (0) does not incise completely the glabella medially; (1) creates a depression such that the anterior glabellar lobe is completely separated from the posterior glabellar region.

12. S3 inclination in relation to SO (Ω angle): (0) 10 – 20°; (1) 20 – 35°.

13. L2 and L3: (0) merge distally; (1) do not merge distally.

14. Ratio between sagittal length of L1 glabellar lobe (L1sl) and glabellar sagittal length, not considering the frontal lobe (gslwfl): (0) 0.35 – 0.45; (1) 0.25 – 0.34; (2) 0.10 – 0.24.

15. Two symmetrical rows of sagittal spines on posterior glabellar region: (0) absent; (1) present.

16. S2 developed as a: (0) shallow depression; (1) deep transverse groove.

17. Number of spines on L2: (0) 0; (1) 1 or 2.

18. Number of spines on L1: (0) 0; (1) 1 or 2.

19. Axial furrows: (0) subparallel posterior and divergent anterior to S3, such that the glabella is mushroom-shaped; (1) evenly divergent from the occipital furrow to the cephalic margin.
20. Incision of cephalic axial furrows: (0) shallow; (1) deep.
21. Width of cephalic axial furrows: (0) narrow; (1) broad.
22. Angle between the axial furrow and the furrow of cephalic posterior border (α angle): (0) 80 – 90°; (1) 75 – 80°; (2) 65 – 75°.
23. Angle between the cephalic posterior border furrow and a line traced from the posterior margin of the axial furrow to the anterior margin of the eyes (β angle): (0) $\geq$ 80°; (1) 65 – 79°; (2) 55 – 64°.
24. S2 and axial furrow: (0) not connected or weakly connected; (1) clearly connected.
25. Ratio between the distance from the posterior margin of the eyes to the axial furrow (dpmeaf) and the maximum glabellar width, not considering the frontal lobe (mgwwfl): (0) between 0 and 0.15; (1) between 0.15 and 0.25.
26. Anterior margin of the eyes: (0) connected to the axial furrow; (1) not connected to the axial furrow.
27. Ratio between the maximum exsagittal length of eyes (mele) and the glabellar sagittal length (gsl): (0) 0.3 – 0.4; (1) 0.2 – 0.3.
28. Size of eyes: (0) large, with their anterior ends opposite S3 and their posterior ends opposite S1; (1) small, with their anterior ends opposite S3, and their posterior ends opposite the middle of L2 or S2 (exsag.).
29. Incision of occipital furrow medially: (0) weakly incised; (1) deeply incised.
30. Elevation of occipital ring: (0) above or equal to L1 and L2; (1) below L1 and L2.
31. Elevation of occipital ring: (0) below or equal to L1 and L2; (1) rising well above entire glabella.
32. Anterior margin of lateral border: (0) curves sharply posteriorly; (1) flexes laterally such that it is roughly parallel to the cephalic posterior border, and at a point distal to the eyes it curves sharply posteriorly.
33. Outline of cephalic margin in dorsal view: (0) semicircular; (1) rounded with medial bulge.
34. Angle between a straight line traced adjacent to the lateral genae (from the contact with the cephalic posterior furrow) and a line traced from the anterior part of

the genae (from the contact of the axial furrow) in direction to the medial-posterior part of the genae (γ angle): (0) 110 – 130°; (1) 130° – 160°.

35. Shape of the genal spine: (0) blunt or blade-like; (1) long.

36. Width of the genal spine (ratio between the spine base width and pygidial width): (0) broad (more than 0.1); (1) slender (less than 0.1).

37. Number of spines on thoracic axial rings: (0) 0; (1) 4 or 5.

38. Pygidial pleural profile in lateral view: (0) arched steeply down from the axial ring; (1) with broad, gently declined proximal region.

39. Dorsoventral height of the pygidium: (0) gradually decreases posteriorly; (1) sharply reduces posterior to the posteriormost apodemal ring.

40. Ratio between maximal sagittal pygidial length (mspl) and maximal transverse pygidial width (mtpw): (0) 0.80 – 1.0; (1) 0.60 – 0.80.

41. Ratio between maximal transverse pygidial axial width (mtpaw) and maximal transverse pygidial width (mtpw): (0) 0.35 – 0.45; (1) 0.25 – 0.35.

42. Ratio between maximal transverse pygidial axial width (mtpaw) and maximal pygidial axial exsagittal length (mpael): (0) 0.45 – 0.65; (1) 0.65 – 0.80.

43. Pygidial shape: (0) elongate, triangular; (1) short, broad.

44. Pygidium axial rings: (0) low and rounded in section (sag.); (1) high and triangular in section (sag.).

45. Shape of the pygidial terminus: (0) rounded; (1) triangular.

46. Spine on the pygidial terminus (ratio between the spine length and pygidial length): (0) absent; (1) short (less than 0.1); (2) long (more than 0.1).

47. Upturn on the terminal pygidium spine: (0) absent; (1) present.

48. Prosopon: (0) smooth to stippled with granules; (1) covered with coarse tubercles; (2) covered with spines.

Appendix 2. Taxon-character matrix of morphological data with 18 taxa and 48 characters. The absence of data is indicated as "?".

Taxon / Character	1	2	3	4	5	6	7	8	9	10	11	12	13	14	15	16	17	18	19	20	21	22	23	24	25	26	27	28	29	30	31	32	33	34	35	36	37	38	39	40	41	42	43	44	45	46	47	48
Kozlowskiaspis (*K.*) *superna*	0	0	-	0	0	0	0	0	0	0	0	0	0	0	0	0	0	0	0	0	0	0	0	0	0	0	0	0	0	0	0	0	0	0	0	0	0	0	0	0	0	0	0	0	0	0	0	0
Wolfartaspis cornutus	1	0	-	0	0	1	0	1	0	1	0	1	0	1	0	1	0	0	0	0	0	0	1	0	0	0	0	0	1	0	1	0	0	?	0	0	0	1	1	0	0	0	1	1	1	2	1	0
Malvinocooperella pregiganteus	1	0	-	0	1	1	0	1	0	1	1	1	0	1	0	1	0	0	0	0	0	0	1	0	0	0	1	1	1	1	0	0	1	0	1	1	0	0	1	1	0	1	1	0	1	1	0	0
Clarkeaspis gouldi	1	1	1	0	1	0	1	1	2	1	1	1	0	1	0	1	0	0	0	0	0	0	?	0	0	?	?	1	0	0	1	0	1	?	?	?	0	?	?	?	?	?	?	?	?	?	?	0
Clarkeaspis padillaensis	1	1	1	0	1	0	0	1	2	1	1	1	0	1	0	1	0	0	0	0	0	1	1	0	0	0	1	1	1	0	1	1	1	0	?	?	0	?	?	?	?	?	?	?	?	?	?	0
Metacryphaeus boulei	1	0	-	1	1	1	1	1	2	1	1	1	0	2	1	1	1	1	1	1	1	1	1	0	1	0	1	1	1	0	1	0	1	1	0	0	1	1	1	0	0	0	1	1	1	1	0	2
Metacryphaeus pujravii	1	1	0	1	1	1	1	0	1	1	0	1	0	2	1	1	1	1	1	0	0	1	1	0	1	0	1	1	0	0	1	0	1	1	1	1	1	1	1	?	0	?	1	1	1	1	0	2
Metacryphaeus giganteus	1	0	-	1	1	1	1	1	2	1	1	1	0	2	0	1	0	0	1	0	1	1	1	0	0	0	0	1	1	0	1	0	1	1	1	1	0	1	0	1	0	1	1	1	1	1	0	0
Metacryphaeus parana	1	0	-	1	1	1	1	0	2	1	1	1	1	1	0	1	0	0	1	0	0	1	1	1	0	0	0	1	0	0	1	0	1	1	1	1	0	1	0	1	0	1	1	1	1	1	0	0
Metacryphaeus tuberculatus	1	1	0	1	1	1	1	1	1	1	1	1	1	1	0	1	0	0	1	1	0	2	2	1	1	1	1	1	1	0	1	0	1	1	0	0	0	1	0	1	0	0	0	1	0	0	0	1
Metacryphaeus convexus	1	0	-	1	1	1	0	0	1	1	1	0	1	2	0	1	0	0	1	1	1	1	1	1	0	0	1	1	1	1	0	0	1	1	?	?	0	1	1	?	?	?	1	1	1	1	0	0
Metacryphaeus curvigena	1	0	-	1	0	1	0	0	0	1	1	0	1	2	0	1	0	0	1	1	1	2	1	1	0	0	0	1	1	0	1	1	1	0	1	0	0	1	0	0	0	0	0	1	1	2	0	0
Metacryphaeus branisai	1	0	-	1	0	1	1	0	1	1	1	1	0	2	0	1	0	0	1	0	0	2	1	0	0	0	?	1	1	0	1	1	1	0	0	0	0	?	?	?	?	?	?	?	?	?	?	0
Metacryphaeus australis	1	0	-	1	1	1	1	1	0	1	1	1	0	1	0	1	0	0	1	0	0	2	1	0	0	0	0	1	0	0	0	0	1	?	0	0	0	1	?	1	1	0	1	1	0	0	0	0
Metacryphaeus caffer	1	1	0	1	1	1	1	?	0	1	1	1	0	1	0	1	0	0	1	0	0	2	1	0	0	0	0	1	0	?	?	0	1	?	0	0	0	1	1	1	1	0	1	1	0	0	0	0
Metacryphaeus kegeli	1	?	?	1	1	1	1	1	0	1	1	1	1	2	0	1	0	0	1	1	1	2	2	1	1	1	?	1	1	?	?	?	1	?	?	?	0	1	?	0	?	0	1	1	0	0	0	0
Metacryphaeus meloi	1	1	0	1	1	1	1	0	2	1	1	0	1	1	0	1	0	0	1	0	0	2	2	1	1	1	0	1	0	0	1	0	1	1	0	0	0	1	1	1	0	0	0	1	0	0	0	0
Metacryphaeus allardyceae	1	1	0	1	1	1	1	0	1	1	1	1	0	1	0	1	0	0	1	0	0	1	1	0	1	0	0	1	0	0	0	0	1	1	?	?	0	1	?	1	0	0	1	?	1	1	0	0
